# Dynamic evolution of Day-To-Day Temperature fluctuations and population exposure on a global scale

**DOI:** 10.1371/journal.pone.0333887

**Published:** 2025-11-14

**Authors:** Hongju Chen, JianPing Yang, Chunping Tan, Jianqiang Wang, Xingran Cai

**Affiliations:** 1 School of Traffic and Transportation, Lanzhou Jiaotong University, Lanzhou, China; 2 State Key Laboratory of Cryospheric Science, Northwest Institute of Eco-Environment and Resources, Chinese Academy of Sciences, Lanzhou, China; 3 Institute for Disaster Management and Reconstruction, Sichuan University–The Hong Kong Polytechnic University, Chengdu, China; 4 Geography Science Institute, Shanxi Normal University, Taiyuan, China; Wuhan University of Technology, CHINA

## Abstract

Understanding the spatiotemporal variations in Day-to-Day Temperature Difference (DTD) and the associated population exposure is essential for evaluating the impacts of climate change on human health, ecosystems, and socio-economic systems. This study offers a comprehensive global analysis of the spatiotemporal patterns of DTD and its interaction with population exposure. Using the CPC Global Unified Gauge-Based Analysis of Daily (CPC GU-GDAD) dataset alongside the LandScan global population data, it evaluates global DTD dynamics and exposure trends from 2000 to 2022. Furthermore, the relative contributions of climatic factors, demographic shifts, and their interactions to changes in exposure levels are systematically quantified. The results demonstrate a pronounced hemispheric asymmetry in DTD distribution, with higher values concentrated in the Northern Hemisphere and a latitudinal peak near 60°N. During the study period, a significant decline in DTD was observed globally, with trends of −0.055°C/decade for daily maximum temperatures and −0.042°C/decade for daily minimum temperatures, indicating an overall stabilization of short-term temperature variability. In contrast, population exposure to DTD exhibited a substantial upward trajectory, increasing annually by 2064 People·°C for maximum temperatures and 1648 People·°C for minimum temperatures, predominantly across the Northern Hemisphere (People·°C: the product of the population size and DTD, used to express the exposure of the population to temperature variability). Furthermore, although global Day-to-Day Temperature variability has declined, population exposure has continued to increase, suggesting that the potential risks associated with temperature fluctuations may persist or intensify, pending further empirical investigation. These findings may support adaptation strategies in urban planning, public health, and climate resilience, particularly in regions with strong DTD variability.

## 1. Introduction

Global climate change remains one of the most significant ecological challenges facing the world today. According to the Sixth Assessment Report of the Intergovernmental Panel on Climate Change (IPCC) [1], the global surface temperature increased by 1.09°C (ranging from 0.95°C to 1.20°C) between 2011 and 2020 compared to the period from 1850 to 1900. Consequently, climate change has become a focal point of global research efforts. Scientists have extensively investigated climate change and its underlying causes across various temporal and spatial scales. Numerous studies have examined the spatiotemporal variations of meteorological elements at annual and monthly timescales [2–5]. Building on these investigations, efforts have been made to delineate climate zones based on observed variations in meteorological parameters [6–9]. Moreover, researchers have employed climate model simulations to project future spatiotemporal changes in climate variables [10–12].

Among the various indicators of climate change, Day-to-Day Temperature Difference (DTD) is considered one of the most direct measures and have been studied since the late 19th and early 20th centuries [13]. Since 1990, only about 20 related articles has been published in English or Chinese meteorological journals, most of which focus on regional characteristics and long-term evolution, while comprehensive analyses of global-scale, multi-timescale variability and potential driving mechanisms remain lacking [14]. Nevertheless, DTD plays a crucial role in understanding the impact of climate change on human activities and ecosystems. Extensive research has demonstrated the significant influence of DTD on public health [14–18], with its effects on public morbidity and mortality well-established through studies at the community [19,20], national [21], and global levels [14]. Meanwhile, researchers have identified a diverse range of impacts associated with DTD, encompassing political and economic activities [7,22], species extinction risk [23], ecosystem functioning [24], food security [25], and extreme temperature events [26]. Furthermore, it is worth noting that DTD has extensive applications in climatology. It can be used to identify discordant signals in station observation sequences [27], to determine the observation period of temperature data [28], and to identify the distribution patterns of local temperature sequences [29]. The indicative significance of DTD for local climate can be used to detect urbanization effects [30]. In addition, the relationship between DTD and weather systems can be used to assess the validity of model results [31]. Understanding the spatiotemporal characteristics of DTD and its population exposure is essential for assessing climate systems’ evolution and societal implications in global climate change. As a key indicator of climate variability, DTD fluctuations may potentially affect public health, agricultural productivity, ecosystem stability, and energy demand. Therefore, a comprehensive investigation into the annual and seasonal trends of DTD, along with its spatial distribution and population exposure across different regions and seasons, is crucial for enhancing climate forecasting capabilities, informing adaptation strategies, and optimizing public health and urban management policies.

The CPC Global Unified Gauge-Based Analysis of Daily (CPC GU-GDAD) dataset is widely recognized as a valuable resource for climate change research and meteorological data assessment [32,33]. In this study, the CPC GU-GDAD dataset is utilized to systematically analyze the spatiotemporal characteristics of Day-to-Day Temperature Difference (DTD) on global and regional scales, covering both annual and seasonal variations from 2000 to 2022. Furthermore, to evaluate the societal implications of DTD variations, this study integrates high-resolution LandScan global population data to examine population exposure to DTD across different regions and seasons. Despite the recognized importance of DTD in understanding climate variability, few studies have systematically assessed its long-term global trends and the associated population exposure risks. Existing research has primarily focused on regional patterns or seasonal variations, often overlooking fine-scale daily fluctuations at the global level. This study addresses this gap by conducting a comprehensive global-scale analysis of DTD changes and quantifying the evolving population exposure risks under decreasing DTD conditions. By revealing the paradoxical trend of more stable daily temperatures coupled with increasing exposure threats, this research provides new insights into climate risk evolution. By investigating these relationships, this study aims to offer critical insights into the spatiotemporal evolution of DTD under global warming and to inform climate adaptation policies, public health risk management, and urban resilience planning.

## 2. Data and method

### 2.1. Datasets and sources

The Climate Prediction Center’s gauge-based analysis of the global daily minimum and maximum temperature and precipitation product (CPC-Global datasets) was initiated by the Climate Prediction Center of the National Oceanic and Atmospheric Administration (NOAA: http://www.cpc.ncep.noaa.gov/). The product is available on 0.5° latitude/longitude grids over the entire global land area from 2000 to 2022 (Table 1).

**Table 1:** Description of data and their sources.

Data Types	Spatial resolution	Temporal Resolution	Obtaining method
CPC-Global datasets	0.5° × 0.5°	2000-2022(daily)	http://www.cpc.ncep.noaa.gov/
LandScan Global Population	0.0083° × 0.0083°	2000-2022(annual)	https://landscan.ornl.gov/

LandScan Global is a high-resolution global population distribution dataset developed by the Oak Ridge National Laboratory (ORNL) of the U.S. Department of Energy. This dataset combines geospatial sciences, remote sensing technology, and machine learning algorithms, aiming to provide the highest resolution global population distribution data. It includes population data in a 30-arcsecond grid format for years 2000-2022 (Table 1). This study utilizes this data to research population exposure (https://landscan.ornl.gov/).

**Fig 1:**
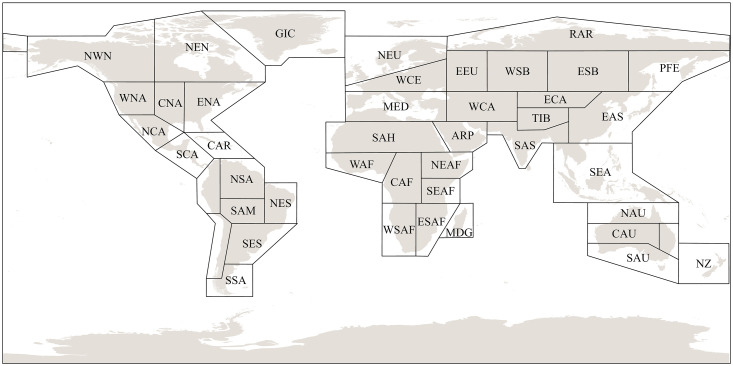
AR6-WGI Reference Region Division Standards.

The AR6-WGI Reference Region Division Standard was established by Working Group I (WGI) of the Sixth Assessment Report (AR6) on Climate Change to delineate and define reference regions in global climate change research. In this study, the Earth’s surface DTD is used for comparison, analysis, and discussion [1]. As shown in Fig 1.

(IPCC AR6 WGI(Intergovernmental Panel on Climate Change,) reference regions: North America: NWN (North-Western North America, NEN (North-Eastern North America), WNA(Western North America), CNA (Central North America), ENA (Eastern North America), Central America: NCA (Northern Central America), SCA (Southern Central America), CAR (Caribbean), South America: NWS (North-Western South America), NSA (Northern South America), NES(North-Eastern South America), SAM (South American Monsoon), SWS (South-Western South America), SES (South-Eastern South America), SSA (Southern South America), Europe: GIC (Greenland/Iceland), NEU (Northern Europe), WCE (Western and Central Europe), EEU (EasternEurope), MED (Mediterranean), Africa: MED (Mediterranean), SAH (Sahara), WAF (Western Africa), CAF (Central Africa), NEAF (North Eastern Africa), SEAF (South Eastern Africa), WSAF (West Southern Africa), ESAF (East Southern Africa), MDG (Madagascar), Asia: RAR (Russian Arctic), WSB (West Siberia), ESB (East Siberia), RFE (Russian Far East), WCA (West Central Asia), ECA (East Central Asia), TIB (Tibetan Plateau), EAS (East Asia), ARP (Arabian Peninsula), SAS (South Asia), SEA (South East Asia), Australasia: NAU (Northern Australia), CAU (CentralAustralia), EAU (Eastern Australia), SAU (Southern Australia), NZ (New Zealand), Small Islands: CAR (Caribbean), PAC (Pacific Small Islands);Source: IPCC AR6 WGI reference regions; Gutiérrez et al., 2021 [34]).

The geographic boundary data employed for map visualizations were obtained from the IPCC AR6 WGI reference region shapefiles, which are made available under the Creative Commons Attribution 4.0 International License. All maps presented in this study were generated by the authors using Python (matplotlib and cartopy) based on the processed gridded datasets and the corresponding vector boundaries. Details of the dataset and its methodology can be found in Gutiérrez et al. (2021) [34].

### 2.2. Methods

Based on the availability of CPC data and LandScan Global, the study period was determined to be from 2000 to 2022. The period 2000–2022 was selected based on the high availability and improved quality of both meteorological and population datasets during this time, ensuring data reliability and consistency for long-term global trend analysis. The CPC data was first used to identify the spatiotemporal distribution of DTD globally throughout the year and across different seasons. Then, LandScan Global was integrated to analyze the population exposure to DTD worldwide. Additionally, the contribution of various factors to the changes in exposure levels was analyzed and discussed.

#### 2.2.1. Identification of DTD.

The Day-to-Day Temperature Difference (DTD) is calculated by averaging the absolute difference between every two adjacent days for a given period [35]. DTD was calculated as Eq. 1


$${\text{DTD}}_{(\text{j})}=\sum_{\text{i}}\left|{\text{T}}_{\text{i}+\text{1}}-{\text{T}}_{\text{i}}\right|/\left(\text{N}-\text{1}\right)$$ (1)

Where j represents the year (2000–2022); T represents the daily maximum or minimum temperature; i represents the day of the year. DTD (℃) is calculated based on the absolute daily temperature difference; N represents the total number of days in the given period (e.g., a year or season) for which the DTD is calculated. A higher DTD value indicates greater diurnal temperature instability, while a smaller value indicates greater stability.

#### 2.2.2. Factors influencing changes in population exposure.

In this study, population exposure (E) is defined at the grid level as the product of DTD (D) and population (P) within each grid cell, expressed in people·℃. Thus, changes in population exposure under DTD are determined by variations in both climatic and demographic factors. These changes can be decomposed into three contributing factors [36]:

①Demographic factor (constant DTD intensity, varying population size).
②Climatic factor (varying DTD intensity, constant population size).
③Combined climatic and demographic factor (both DTD intensity and population size vary).

The change in population exposure (E) can be expressed as:


(D+∆D)× (P+∆P)−D×P=∆D×P+D×∆P +∆D×∆P (2)

The contribution rate of the climatic factor:


$$\frac{\Delta \text{D}\times \text{P}}{\left(\text{D}+\Delta \text{D}\right)\times \;\left(\text{P}+\Delta \text{P}\right)-\text{D}\times \text{P}}\times \text{100}\%$$ (3)

The contribution rate of the demographic factor:


$$\frac{\text{D}\times \Delta \text{P}}{(\text{D}+\Delta \text{D})\times \;(\text{P}+\Delta \text{P})-\text{D}\times \text{P}}\times \text{100}\%$$ (4)

The contribution rate of the combined climatic and demographic factor:


$$\frac{\Delta \text{D}\times \Delta \text{P}}{(\text{D}+\Delta \text{D})\times \;(\text{P}+\Delta \text{P})-\text{D}\times \text{P}}\times \text{100}\%$$ (5)

where D and P represent the DTD value and population size, respectively; ΔD denotes the given period change in DTD, while ΔP represents the annual change in population size within each grid cell.

This study adopts the year 2000 as the baseline; ΔD × P represents the climatic factor, D × ΔP represents the demographic factor, and ΔD × ΔP represents the combined climatic and demographic factor.

## 3. Result

### 3.1. Analysis of global DTD characteristics

#### 3.1.1. Spatial patterns of global DTD.

**Fig 2:**
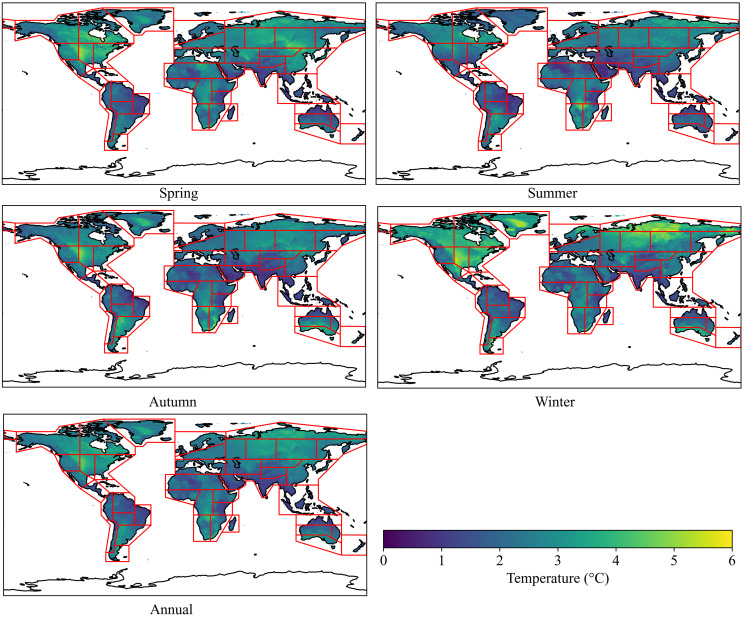
Spatial distribution of multi-year average DTD for daily maximum temperature from 2000 to 2022.

**Fig 3:**
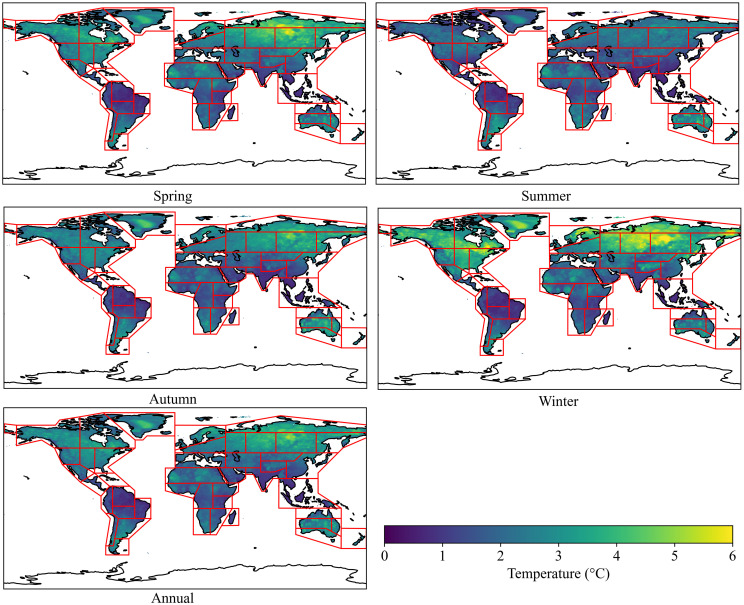
Spatial distribution of multi-year average DTD for daily minimum temperature from 2000 to 2022.

**Fig 4:**
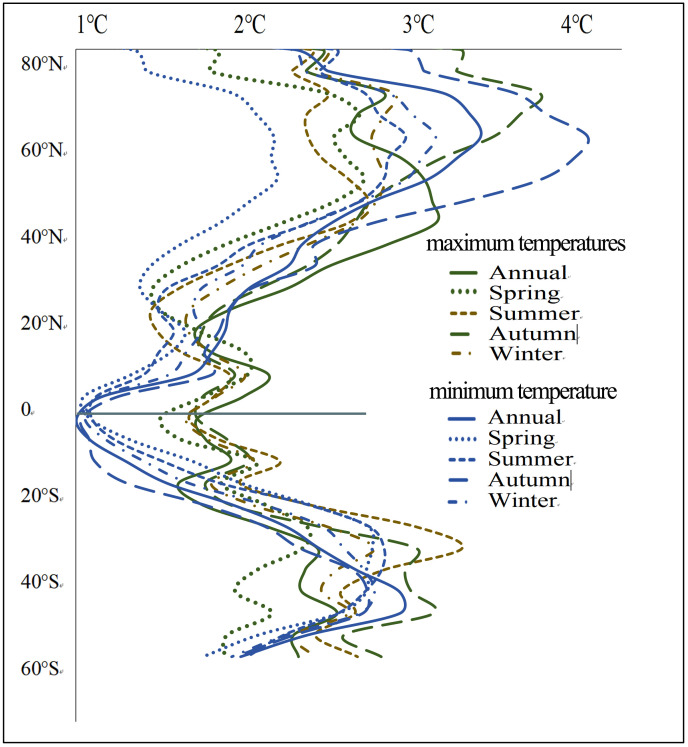
Latitudinal Distribution of Multi-Year Average DTD.

According to Fig 2 and Fig 3, the spatial distribution and intensity of DTD values for daily maximum and minimum temperatures exhibit strong consistency within the same season. Seasonally, the highest DTD values for both daily maximum and minimum temperatures are observed in RAR, ESB, WSB, NEN, NWN, CNA, and WNA, whereas the lowest values occur in SAS, SEA, NSA, and NES. From a latitudinal perspective, the mean DTD values exhibit an asymmetrical distribution between the Northern and Southern Hemispheres. On an annual scale, as well as during autumn and winter, the DTD of both daily maximum and minimum temperatures is significantly higher in the Northern Hemisphere than in the Southern Hemisphere. Additionally, in the Northern Hemisphere, DTD values increase with latitude initially, reach a peak around 60°N, and then decline (Fig 4). In equatorial regions (20°S–20°N), the DTD of daily maximum temperatures is generally higher than that of daily minimum temperatures. However, in high-latitude regions near 60°N, this pattern reverses, with the DTD of daily minimum temperatures exceeding that of daily maximum temperatures, indicating a distinct latitudinal dependence of DTD variability.

Regarding the global mean DTD, on an annual scale, the DTD of daily maximum temperatures (2.42℃) is slightly higher than that of daily minimum temperatures (2.35℃). Seasonally, in spring and autumn, the DTD of maximum temperatures exceeds that of minimum temperatures, whereas in autumn and winter, the DTD of minimum temperatures surpasses that of maximum temperatures. Notably, winter exhibits the highest DTD for minimum temperatures, while summer records the lowest DTD for minimum temperatures, highlighting significant seasonal variability (Table 2).

**Table 2:** Global average DTD from 2000 to 2022(Unit: °C).

	Annual	Spring	Summer	Autumn	Winter
maximum temperatures	2.42	2.52	2.19	2.28	2.71
minimum temperature	2.35	2.50	1.87	2.29	2.78

#### 3.1.2. Temporal variation characteristics of global average DTD.

**Fig 5:**
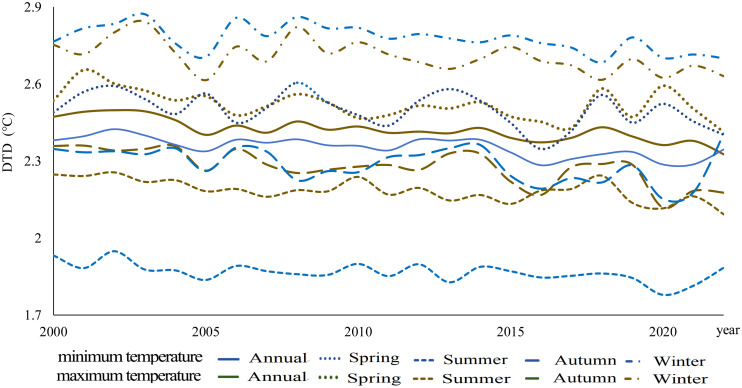
Global average DTD from 2000 to 2022.

Fig 5 illustrates the temporal variation of DTD from 2000 to 2022, covering the annual average DTD and seasonal variations (spring, summer, autumn, and winter) of daily maximum and minimum temperature DTD. Throughout the year and across all seasons, the DTD of daily maximum temperatures remains consistently higher than that of daily minimum temperatures, and both exhibit a significant downward trend. Specifically, the annual decreasing trends for daily maximum and minimum temperature DTD are -0.055℃/10y (95%CI: [-0.0070, -0.0040])and -0.042℃/10y(95%CI: [-0.0060,-0.0025]), respectively. Among the four seasons, autumn exhibits the most pronounced decline in daily maximum temperature DTD, with a rate of -0.074℃/10y(95%CI: [-0.0104, -0.0044]), whereas summer shows the smallest decline in daily minimum temperature DTD, with a rate of -0.031℃/10y(95%CI:[-0.0051, -0.0012]) (Table 3). These findings indicate a seasonally dependent reduction in DTD, with the most substantial decline occurring in autumn for maximum temperatures, while the weakest decline is observed in summer for minimum temperatures.

**Table 3:** Global Average DTD Change Rate from 2000 to 2022 (Unit: °C/10y).

		Annual	Spring	Summer	Autumn	Winter
maximum temperatures	Rate	−0.055 **	−0.045 *	−0.045**	−0.074**	−0.055**
	95%CI	[−0.0070, −0.0040]	[−0.0079, −0.0012]	[−0.0067, −0.0024]	[−0.0104, −0.0044]	[−0.0088, −0.0022]
minimum temperature	Rate	−0.042 **	−0.045 *	−0.031 **	−0.045 *	−0.048 **
	95%CI	[−0.0060, −0.0025]	[−0.0085, −0.0006]	[−0.0051, −0.0012]	[−0.0085, −0.0004]	[−0.0077, −0.0019]

p < 0.01 (**) , p < 0.05 (*) , p: p-value indicate the confidence level, CI: Confidence Interval.

### 3.2. Population exposure under DTD

#### 3.2.1. Spatial distribution characteristics of population exposure.

**Fig 6:**
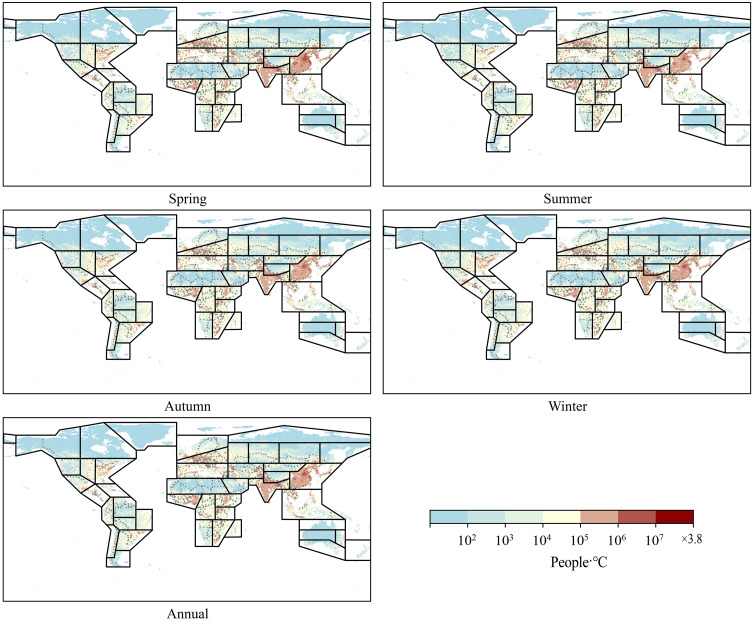
Spatial Distribution of Population Exposure under Daily Maximum Temperature DTD from 2000 to 2022.

**Fig 7:**
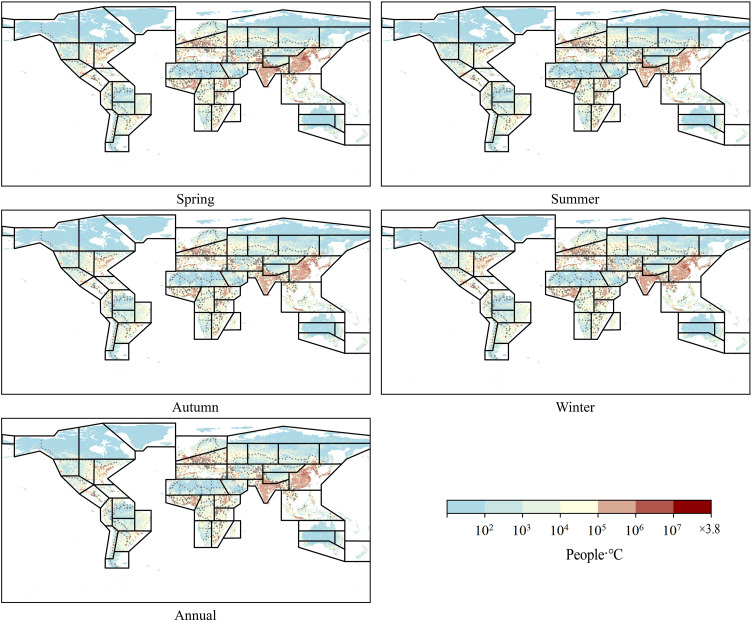
Spatial Distribution of Population Exposure under Daily Minimum Temperature DTD from 2000 to 2022.

**Fig 8:**
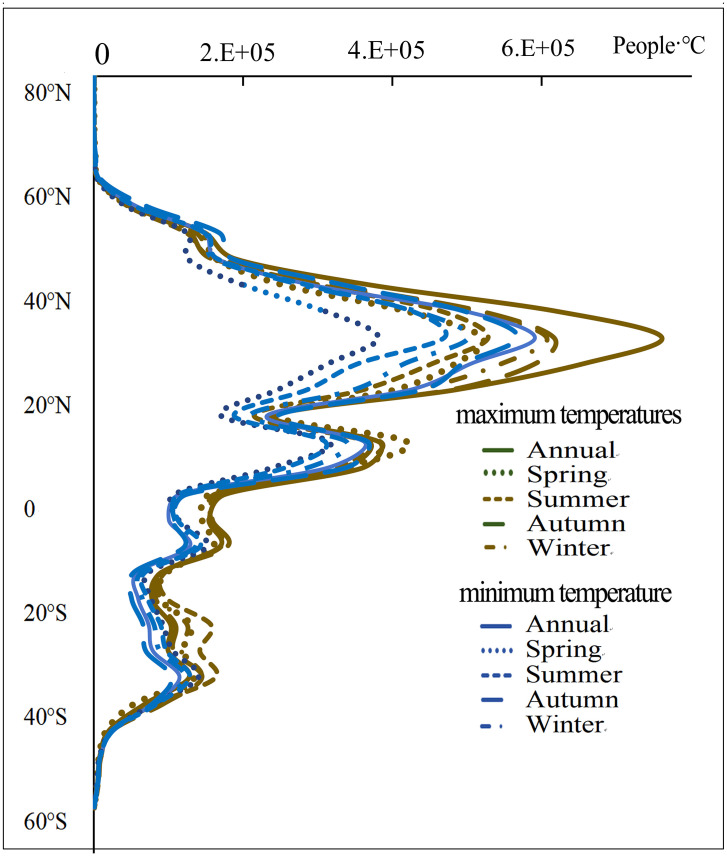
Latitudinal Distribution of Population Exposure under DTD.

According to Fig 6 and Fig 7, population exposure under DTD is influenced by both DTD intensity and population distribution. Given that the spatial patterns and intensity of DTD for daily maximum and minimum temperatures exhibit strong consistency within the same season, the spatial distribution of population exposure under DTD also demonstrates significant coherence. Across annual and seasonal scales, population exposure under daily maximum and minimum temperature DTD is highest in the EAS, SAS, WCE, WAF, WCA, NEAF, and MED regions. From a latitudinal perspective, the multi-year average population exposure under DTD is notably higher in the Northern Hemisphere than in the Southern Hemisphere. Within the latitude range of 5°N–35°N, population exposure under both daily maximum and minimum temperature DTD reaches its peak values. Notably, two maxima are observed near 10°N and 37°N, while a local minimum occurs around 20°N, indicating a non-uniform latitudinal distribution of population exposure (Fig 8). In terms of global total population exposure, across both annual and seasonal timescales, population exposure under daily maximum temperature DTD consistently exceeds that under daily minimum temperature DTD. The highest population exposure is recorded under annual daily maximum temperature DTD, while the lowest exposure is observed under spring daily minimum temperature DTD, with values of 1.5 × 10¹⁰ People·℃ and 9.1 × 10¹⁰ People·℃, respectively. These results highlight the dominant role of both climate conditions and population density in shaping the spatial distribution of population exposure under DTD (Table 4).

**Table 4:** Global Average Population Exposure (Unit: × 10^10^ People·°C).

	Annual	Spring	Summer	Autumn	Winter
maximum temperatures	1.50	1.22	1.22	1.35	1.32
minimum temperature	1.24	9.10	1.07	1.27	1.12

#### 3.2.2. Temporal variation analysis of population exposure.

**Fig 9:**
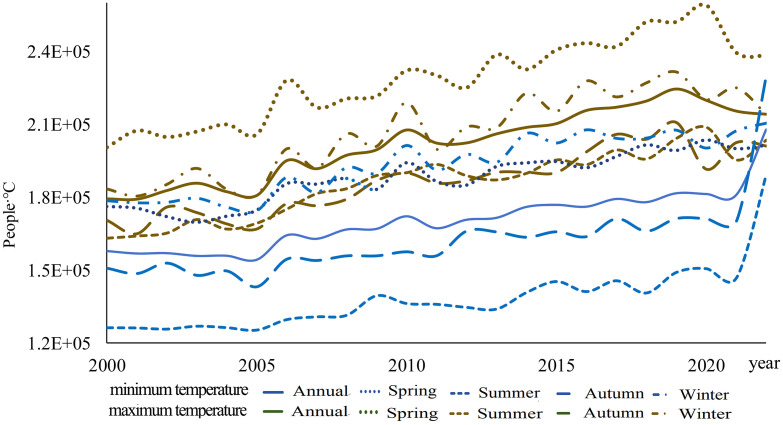
Global Average Population Exposure Change from 2000 to 2022.

Fig 9 illustrates the temporal variation of population exposure under DTD from 2000 to 2022, encompassing the annual average DTD and seasonal variations (spring, summer, autumn, and winter) of population exposure under daily maximum and minimum temperature DTD. Throughout this period, population exposure under daily maximum temperature DTD consistently exceeds that under daily minimum temperature DTD across all seasons and the annual scale, and both exhibit a significant increasing trend. Specifically, the annual increasing trends for population exposure under daily maximum and minimum temperature DTD are 2064 People·℃/year (95% CI: [1770, 2356]) and 1648 People·℃/year(95% CI: [1305, 1990]), respectively. Among the four seasons, Spring shows the most pronounced increase in population exposure under daily maximum temperature DTD, with a rate of 2299 People·℃/year(95% CI: [1863, 2733]). In contrast, Spring exhibits the smallest increase in population exposure under daily minimum temperature DTD, with a rate of 1456 People·℃/year (95% CI: [1206, 1705]) (Table 5).

**Table 5:** Global Average Population Exposure Change Rate from 2000 to 2022(Unit: People·°C/year).

		Annual	Spring	Summer	Autumn	Winter
maximum temperatures	Rate	2064**	2299**	1935**	1796**	2229**
	95%CI	[1770, 2356]	[1863, 2733]	[1640, 2229]	[1449., 2142]	[1728., 2728]
minimum temperature	Rate	1648**	1456**	1631**	1844**	1666**
	95%CI	[1305, 1990]	[1206, 1705]	[1082, 2179]	[1072, 2615]	[1356, 1974]

p < 0.01 (**) , p < 0.05 (*) , p: p-value indicate the confidence level, CI: Confidence Interval.

### 3.3. Analysis of factors influencing population exposure changes

**Fig 10:**
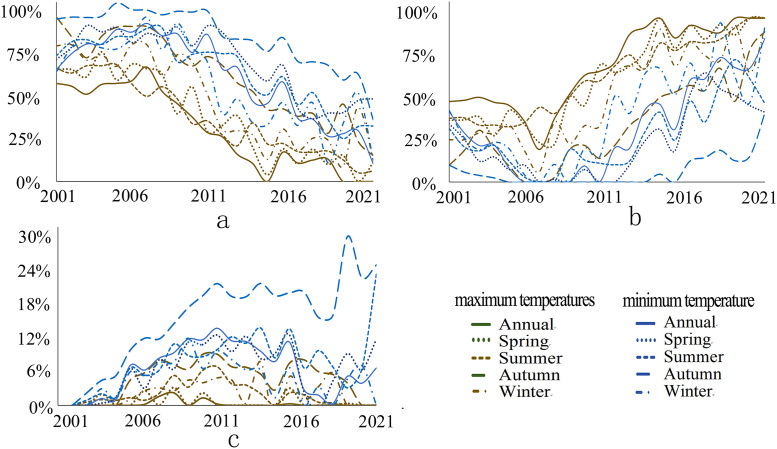
Contribution of Influencing Factors to Population Exposure Changes (a, climate alone; b, population alone; c: the combined effects of climate and population).

The variation in population exposure under DTD is influenced by both climatic factors and changes in population size. Using the year 2000 as the baseline, this study investigates the factors contributing to changes in population exposure from 2001 to 2022. Three scenarios are considered: climatic factors (fixed population, changing DTD), demographic factors (changing population, fixed DTD), and combined climatic and demographic factors (simultaneous changes in both population and DTD). The contribution of each factor to population exposure changes during extreme high-temperature events is analyzed across different periods and regional scales. As shown in the Fig 10, before 2004, changes in population exposure were primarily driven by both climatic and demographic factors, while after 2006, the contribution of climatic factors significantly weakened. In contrast, the influence of demographic factors and the combined effects of climate and population became increasingly prominent. Notably, the contributions of climatic and combined climatic-demographic factors to population exposure changes under daily minimum temperature DTD were significantly higher than those under daily maximum temperature DTD across both annual and seasonal scales. Conversely, the contribution of demographic factors to population exposure changes under daily minimum temperature DTD was consistently lower than that under daily maximum temperature DTD.

## 4. Discussion

### 4.1. Discussion on the evolution of DTD and population exposure under climate change and its potential risks

This study demonstrates that from 2000 to 2022, the global DTD exhibited an overall decreasing trend, with the DTD of daily maximum temperatures declining at -0.055℃/10y and that of daily minimum temperatures at -0.042℃/10y. Among the seasons, autumn experienced the most pronounced decrease in maximum temperature DTD (-0.074℃/10y), whereas summer showed the smallest decline in minimum temperature DTD (-0.031℃/10y). This trend is likely primarily driven by the increasing concentration of atmospheric greenhouse gases due to global warming. The accumulation of greenhouse gases, such as CO₂ and CH₄ [1], enhances the longwave radiation trapping effect at night, reducing nighttime cooling rates and thereby weakening temperature variations between consecutive days, leading to a decrease in DTD. Additionally, the intensified greenhouse effect has resulted in increased atmospheric water vapor content and cloud cover, further stabilizing temperature fluctuations and reducing Day-to-Day Temperature Difference variations, particularly in low-latitude and coastal regions where these effects are more pronounced. Between 2000 and 2022, global population exposure under DTD exhibited a significant upward trend, with exposure levels in the Northern Hemisphere (particularly in East Asia, South Asia, Europe, and North America) far exceeding those in the Southern Hemisphere. The most notable increases in exposure were observed in densely populated regions prone to extreme high temperatures, such as South Asia, Southeast Asia, and sub-Saharan Africa. Population exposure peaked at latitudes near 10°N and 37°N, while at around 60°N, exposure under minimum temperature DTD surpassed that under maximum temperature DTD, indicating that nighttime temperature fluctuations have a greater impact on populations in these regions. Previous studies have highlighted the health risks associated with large DTD. Guo et al. [14] found that greater DTD variability increases the incidence of cardiovascular and respiratory diseases, with elderly individuals and children being particularly vulnerable. Under extreme DTD conditions, mortality rates and disease burdens are likely to intensify. Moreover, the rising population exposure under DTD may result in a series of socioeconomic challenges, including agricultural production instability [25], increased fluctuations in energy demand, and challenges in infrastructure development and urban planning. In high-latitude regions, the increase in minimum temperature DTD may impose greater demands on heating systems and preparedness for extreme cold weather events.

Thus, global climate change is significantly altering the distribution of population exposure under DTD, with far-reaching implications for public health, agricultural production, energy management, and urban infrastructure. Future research should integrate high-resolution meteorological datasets and socioeconomic models to further explore the roles of urbanization, adaptation strategies, and global warming in shaping population exposure under DTD.

### 4.2. Research limitations and future directions

Despite conducting a systematic analysis of global DTD variations and associated population exposure, this study has several limitations that warrant further discussion. One limitation arises from the spatial resolution of the dataset used. The CPC GU-GDAD dataset has a resolution of 0.5° × 0.5°, which may not be sufficient to capture fine-scale temperature variations in regions with complex topography or localized climatic influences. This spatial constraint could affect the accuracy of DTD assessments, particularly in mountainous or urbanized areas. To address this, we have expanded the discussion in the revised manuscript. Future research should explore the use of higher-resolution datasets and conduct regional sensitivity analyses to better characterize local-scale exposure patterns. Another important consideration is the method used to calculate DTD. The analysis in this study primarily focuses on seasonal and annual timescales, without addressing short-term DTD variations. Future research should explore appropriate time windows for quantifying short-term DTD changes, thereby offering a more comprehensive understanding of its spatiotemporal dynamics and potential impacts. Furthermore, the health risks associated with DTD variations may vary significantly across different regions and population groups, such as the elderly, children, and individuals with chronic diseases. However, this study only examines population exposure without incorporating a quantitative assessment of health risks, making it difficult to fully capture the public health implications of DTD changes. Future research should integrate higher-resolution climate datasets and epidemiological models to enhance analytical precision and to further investigate the societal impacts of DTD variations.

## 5. Conclusion

This study systematically analyzed the spatiotemporal variations of global Day-to-Day Temperature Difference (DTD) and their associated population exposure from 2000 to 2022. Furthermore, the contributions of different influencing factors—climatic, demographic, and combined climatic-demographic factors—to changes in population exposure were investigated. The key findings are as follows:

(1) Spatial Distribution Characteristics: Across different statistical periods, DTD values in the Northern Hemisphere were generally higher than those in the Southern Hemisphere. In terms of latitude, DTD peaked around 60°N.
(2) Temporal Trends of DTD: A significant decreasing trend in global DTD was observed from 2000 to 2022, with a decline rate of −0.055℃/10y for daily maximum temperature DTD and −0.042℃/10y for daily minimum temperature DTD. Seasonally, the most pronounced decrease occurred in autumn for maximum temperature DTD (−0.074℃/10y), while the weakest decline was observed in summer for minimum temperature DTD (−0.031℃/10y). These results indicate an overall stabilization of Day-to-Day Temperature fluctuations on a global scale.
(3) Changes in Population Exposure Under DTD: Population exposure under DTD showed a significant upward trend from 2000 to 2022, with annual growth rates of 2064 People·℃/year under daily maximum temperature DTD and 1648 People·℃/year under daily minimum temperature DTD. Spatially, population exposure was significantly higher in the Northern Hemisphere, particularly in East Asia, South Asia, Europe, and North America, compared to the Southern Hemisphere.
(4) Contributions of Climate and Population Factors: The increase in population exposure was primarily driven by a combination of population growth and climate change. Before 2004, population exposure variations were mainly influenced by both climatic and demographic factors. However, after 2006, the contribution of climatic factors significantly weakened, while the influence of demographic factors and the combined effects of climate and population became increasingly prominent.

As an important climate indicator, DTD variations may have profound implications for societal, economic, and ecological systems. This study provides a preliminary assessment of global DTD changes and their population exposure from 2000 to 2022. However, the underlying mechanisms of these changes and their potential impacts remain unclear, necessitating further research to explore their broader implications.
